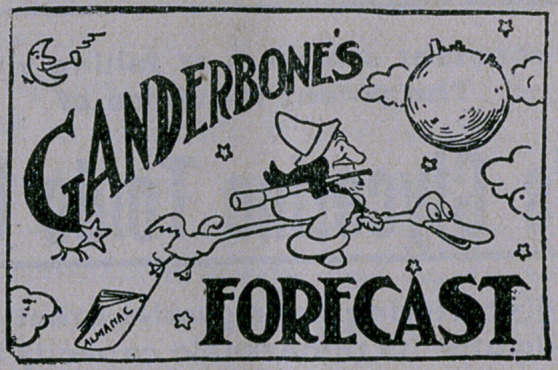# Selected

**Published:** 1909-04

**Authors:** 


### Selected.

**For April.**

(Copyright, 1909, by C. H. Rieth.)

---

The beasts of Uganda were beating retreat, and the slower were trying to stay with the fleet, when a lion came flying aside from the rear with his face mutilated and one shredded ear.

"We thought," said the beasts
As they saw him arrive,

"You stayed back to face him
  And eat him alive!"
But the man-eater only
  Ran faster and whined,
And now and then ventured
  A survey behind.

"Well," said the hippo, who ran as he could, "did you eat him alive, as you boasted you would?" But the lion limped onward with never a word—at least anything that the rest of them heard.

"I'll bet," said the rhino,
  "He bit off his head
And left them to find him
  And bloody and dead!"
Whereat they all laughed,
  And the great lion cried
And licked at the gashes
  All over his hide.

"I see how it happened," the elephant said; "our brother was up in a tree overhead, and when he pounced on him, a stranger to fear, the cruel thorns tore him and shredded his ear!"

They all laughed again,
  And the lion, all red
With blood, only shuddered
  And limped on ahead.
"O, come," they exclaimed
  As they followed with haste,
"We know that you ate him,
  But how did he taste?"

The lion turned round at the top of a rise, and his whiskers were matted with tears from his eyes. "Don't taunt me," he begged, "and I'll tell you my woes." And blood trickled off at the end of his nose.

"Well, do it!" they answered,
  And husky with grief
And fear, he proceeded:
  "My story is brief.
I did lay for Teddy,
  Intending my worst,
And I jumped as I promised—
  But he bit me first."

The rain will patter on the roof, and the colt will buck and dance, the tickled calf will shake his hoof and jubilantly prance; the robin will arise at morn and chase the festive worm, the school-boy will wish chickenpox would spread and end the term, the old fleas will breed other fleas upon the itching pup, and the dirt will fly with radishes and lettuce coming up.

Easter will come on the 11th. It is too early to predict just what the new hats will be like. From the few young ones we have seen the merry widow seems to have married a derby. The issue resembles neither parent. The millions were determined to make something that would keep anyone from trimming her old hat over, and they did it.

Primp, sisters, primp, primp with care,
Use your own and other hair,
A white stuffed rat for a light fluffed hat,
A well-draped rat for a bell-shaped hat,
A small, lean rat for a tall green hat.
Primp, sisters, primp, primp with care,
Primp for the coming Easter fair.

## Figures and Tables

**Figure f1:**